# A Manual of Syphilis and the Venereal Diseases

**Published:** 1896-12

**Authors:** 


**A Manual of Syphilis and the Venereal Diseases.** By JAMES NEVINS HYDE, M.D., and FRANK H. MONTGOMERY, M.D. Pp. 1—8, 17—618. Philadelphia: W. B. Saunders. 1895.

In this book the authors lay before their readers in a clear and concise form our present knowledge of the study and treatment of syphilis and the venereal diseases. Great care has been taken throughout to exclude from the text all controversial points and theories. We would call special attention to the sections on "Syphilis in Relation with the Family and Society" and "Hypochondriasis," as containing many valuable hints for the guidance of the young practitioner. The question of the insurability of the lives of those affected with syphilis is well considered. The practice of irrigation in acute urethritis, so much vaunted by some American authors, is not recommended.

The book is printed in clear type, the illustrations are on the whole good, and include an excellent coloured plate of Ducrey's bacillus. We are convinced that the work will be appreciated by the student and busy practitioner.

